# Alphavirus Replicon DNA Vectors Expressing Ebola GP and VP40 Antigens Induce Humoral and Cellular Immune Responses in Mice

**DOI:** 10.3389/fmicb.2017.02662

**Published:** 2018-01-10

**Authors:** Shoufeng Ren, Qimei Wei, Liya Cai, Xuejing Yang, Cuicui Xing, Feng Tan, Jianmei W. Leavenworth, Shaohui Liang, Wenquan Liu

**Affiliations:** ^1^Department of Human Parasitology, Wenzhou Medical University, Wenzhou, China; ^2^Institute of Pathogen and Immunology, Wenzhou Medical University, Wenzhou, China; ^3^Department of Laboratory Medicine, The First Affiliated Hospital of Zhejiang Chinese Medical University, Hangzhou, China; ^4^Department of Neurosurgery, University of Alabama at Birmingham, Birmingham, AL, United States; ^5^Department of Microbiology, University of Alabama at Birmingham, Birmingham, AL, United States

**Keywords:** Ebola virus, GP and VP40 antigens, virus-like particles, SFV replicon vector, immune response, vaccine

## Abstract

Ebola virus (EBOV) causes severe hemorrhagic fevers in humans, and no approved therapeutics or vaccine is currently available. Glycoprotein (GP) is the major protective antigen of EBOV, and can generate virus-like particles (VLPs) by co-expression with matrix protein (VP40). In this study, we constructed a recombinant Alphavirus Semliki Forest virus (SFV) replicon vector DREP to express EBOV GP and matrix viral protein (VP40). EBOV VLPs were successfully generated and achieved budding from 293 cells after co-transfection with DREP-based GP and VP40 vectors (DREP-GP+DREP-VP40). Vaccination of BALB/c mice with DREP-GP, DREP-VP40, or DREP-GP+DREP-VP40 vectors, followed by immediate electroporation resulted in a mixed IgG subclass production, which recognized EBOV GP and/or VP40 proteins. This vaccination regimen also led to the generation of both Th1 and Th2 cellular immune responses in mice. Notably, vaccination with DREP-GP and DREP-VP40, which produces both GP and VP40 antigens, induced a significantly higher level of anti-GP IgG2a antibody and increased IFN-γ secreting CD8^+^ T-cell responses relative to vaccination with DREP-GP or DREP-VP40 vector alone. Our study indicates that co-expression of GP and VP40 antigens based on the SFV replicon vector generates EBOV VLPs *in vitro*, and vaccination with recombinant DREP vectors containing GP and VP40 antigens induces Ebola antigen-specific humoral and cellular immune responses in mice. This novel approach provides a simple and efficient vaccine platform for Ebola disease prevention.

## Introduction

Ebola virus (EBOV), an enveloped RNA virus, belongs to the genus *Ebolavirus* in the Filoviridae family (Holmes et al., 2016). There are five species of EBOV, including Zaire virus (ZEBOV), Sudan virus (SEBOV), Taï Forest virus (TEBOV), Bundibugyo virus (BEBOV), and Reston virus (REBOV). The former four EBOV are known to cause severe hemorrhagic fever in humans, with case fatality rates of up to 90% (Feldmann et al., 2003; Holmes et al., 2016). Since the first case reported in 1976, there have been numerous outbreaks of Ebola severe hemorrhagic fevers in Africa (Feldmann et al., 2003), with the largest epidemic occurring in West Africa from 2013 to 2016, during which there were almost 30,000 infections and more than 11,000 deaths (Baize et al., 2014; Holmes et al., 2016). Currently, a range of potential treatments including antibody therapies and drug therapies are being evaluated, however, no licensed EBOV vaccine is available for pre- or post-exposure treatment (Marzi and Feldmann, 2014; Reynolds and Marzi, 2017).

The EBOV possesses a characteristic threadlike appearance and a negative-sense single-strand RNA genome of approximately 19-kilobases coding seven structural proteins: VP24, VP30, VP35, nucleoprotein (NP), the large protein (L), matrix protein (VP40), and glycoprotein (GP) (Messaoudi et al., 2015). The GP protein forms spikes on the Ebola virion surface, which is responsible for receptor binding and membrane fusion (Licata et al., 2004; Mohan et al., 2015), whereas the VP40 protein plays an important role in particle morphogenesis and budding (Noda et al., 2002; Liu et al., 2010). Induction of anti-GP antibodies by the recombinant EBOV vaccine is necessary to provide protection against EBOV infection in nonhuman primates (Blaney et al., 2013; Pyankov et al., 2015). The important contribution of GP antibodies to protection is further supported by the passive transferring of neutralizing monoclonal antibodies in cynomolgus macaques, which results in complete survival from EBOV challenge (Qiu et al., 2012). Therefore, GP protein (alone or in combination with VP40) is chosen as the primary immunogen in the majority of vaccine candidates against EBOV infection, such as attenuated recombinant EBOV vaccines (Papaneri et al., 2012), DNA vaccines (Martin et al., 2006), and virus-like particles (VLPs) vaccines (Warfield et al., 2003, 2007; Sun et al., 2009).

Co-expression of GP and VP40 proteins leads to incorporation of Ebola VLPs displaying similar structural characteristics and antigenic epitopes to the parental virus (Noda et al., 2002; Licata et al., 2004). Ebola VLPs have been produced in both mammalian and insect cell expression systems, exhibiting full protection in rodents and nonhuman primates after immunization (Warfield et al., 2003; Sun et al., 2009). However, the progression of Ebola VLPs toward clinical trials has been hampered by manufacturing hurdles, which include inefficient transfection, poor reproduction, and low yield in the mammalian expression system (Warfield and Aman, 2011), as well as obvious differences in the GP glycosylation pattern in insect cell–derived VLPs compared with mammalian cell-derived VLPs (Sun et al., 2009; Mohan et al., 2015).

Recently, several replicating viral vectors, such as Vesicular stomatitis virus (VSV), Rabies virus, and Alphavirus, have been developed into recombinant viruses expressing EBOV GP antigen that induce protective immune responses in nonhuman primates (Blaney et al., 2013; Pyankov et al., 2015; Williams et al., 2015). Semliki Forest virus (SFV), a member of Alphavirus genus, infects a wide variety of cell types from both mosquitoes and mammals, but generally does not cause disease in humans (Leung et al., 2011). SFV replicon-based DREP vector contains two open reading frames (ORFs). The first ORF encodes a replicase complex which directs replication and amplification of the viral genome. The second ORF encodes a foreign antigen, which can be produced continuously in a large amount under the control of the replicase complex and 26S subgenomic promoter (Leitner et al., 2003). Therefore, the DREP vector can induce higher humoral and cellular immune responses compared with conventional DNA vaccines, such as pCMV vector (Berglund et al., 1998; Nordstrom et al., 2005).

In this study, we constructed the recombinant SFV replicon DNA vectors DREP-GP and DREP-VP40 to express the Zaire EBOV (2014 epidemic strain) GP and VP40 proteins, respectively. The assembly of VLPs was confirmed by co-transfection with DREP-GP and DREP-VP40 *in vitro*. The humoral and cellular immunological characterizations were studied after vaccination with DREP-based Ebola GP and VP40 vectors by electroporation into BALB/c mice.

## Materials and Methods

### Cells and Antibodies

The human embryonic kidney 293 cell line was maintained in Dulbecco’s Modified Eagle medium (DMEM, Gibco) supplemented with 10% fetal bovine serum, penicillin (100 U/ml), streptomycin (100 μg/ml), and L-glutamine (2 mM). Cells were maintained at 37°C in 5% CO_2_. Mice monoclonal antibody to ZEBOV VP40 was purchased from Santa Cruz Biotechnology (United States). Rabbit polyclonal antibodies to ZEBOV GP and β-actin were purchased from Immune Technology (United States). The recombinant ZEBOV GP and VP40 proteins were purchased from Immune Technology (United States) and Sino biological (China), respectively.

### Ethics Statement

This study was carried out in accordance with the recommendations of National Institutes of Health Guidelines for the Care and Use of Experimental Animals. The protocol was approved by the Laboratory Animal Ethics Committee of Wenzhou Medical University (Permit number: wydw2015-0027), Zhejiang, China.

### Construction of SFV Replicon Vectors Expressing GP and VP40

The cDNA sequences encoding full-length wild-type GP (GenBank Accession No. AHX24667.2) and VP40 (GenBank Accession No. AHX24648.1) of ZEBOV (2014) were synthesized and codon optimized (GeneScript, Nanjing, China). The DREP-eGFP vector was kindly provided by Dr. Peter Liljeström, and the genes of GP and VP40 were individually cloned into the downstream of the 26S subgenomic promoter to replace the eGFP sequences. The restriction enzymes, *Xma* I and *Spe* I, were used in the cloning and restriction enzyme digestion. The recombinant DREP-GP and DREP-VP40 vectors were confirmed by DNA sequencing (Genomics, Shanghai, China). Plasmids were purified using Qiagen Plasmid Maxi Kits (Qiagen, United States), and stored at -20°C until use. The DNA concentration was determined by using a Du530 spectrophotometer (Beckman, Germany) at OD_260_.

### Protein Expression and Detection

A total of 293 cells were transfected individually with the recombinant plasmids DREP-GP, DREP-VP40, or DREP-GP+DREP-VP40 (plasmid mixture with equal amounts of DREP-GP and DREP-VP40) by using the *Trans*IT-LT1 reagent (Mirus, United States) according to the manufacturer’s instructions. A total of 293 cells were used as the mock control. Transfected cells were incubated at 37°C under 5% CO_2_ for 48 h prior to harvesting. The protein samples were separated through 10% SDS–PAGE and transferred onto polyvinylidene difluoride membranes (PVDF, Millipore). The membranes were blocked in a solution of Tris-buffered saline containing 5% non-fat dry milk and 0.05% Tween 20 and subsequently probed with the indicated antibodies specific for ZEBOV anti-GP or anti-VP40 antibodies. β-actin was taken as the internal control. Antigens were visualized with an alkaline phosphatase-conjugated anti-mouse or anti-rabbit IgG antibody (ABI) according to the manufacturer’s instructions.

Transfected 293 cells were analyzed by the immunofluorescence microscopy as described previously (Mohan et al., 2015). Briefly, samples were fixed with 4% paraformaldehyde at 37°C for 15 min, then permeabilized with 0.5% Triton X-100 at 37°C for 15 min. After blocking at 37°C for 30 min, samples were incubated with the primary antibodies at 37°C for 2 h. After washing with phosphate-buffered saline (PBS) containing 3% bovine serum albumin for 5 times, secondary antibodies labeled with Alexa Fluor 488/568 (ABI) (1:1000) were used at 37°C for 1 h. Samples were analyzed with a Leica SP5 confocal microscope using an NICKON.

### Electron Microscopy Analysis

A total of 293 cells on 6-well tissue culture dishes were transfected with DREP-GP, DREP-VP40, or DREP-GP+DREP-VP40 plasmids. Transfected cells were harvested and centrifuged at 1,500 rpm in a 1.5 mL EP tube for 10 min to form a loose pellet. Samples were then prepared for transmission electron microscopy (TEM); chemically fixed and osmium-stained cells were processed for flat embedding and ultrathin sections. The samples were stained with uranyl acetate and lead citrate according to standard protocols and examined with a Philips CM-100 Transmission Electron Microscope. For negative staining, 12 ml culture supernatant of transfected 293 cells were collected and clarified at 3,000 rpm for 10 min to remove the cell. The supernatant was overlaid on a 20% sucrose in PBS buffer (pH 7.2), and centrifuged at 39,800 rpm for 2 h at 4°C. The pellets were resuspended in 500 μL of PBS buffer and added onto a Formvar-coated copper grid, stained with 2% phosphotungstic acid solution. The samples were examined with Philips Electron Microscope at 80 kV.

### Immunization of Mice

The 6- to 8-week-old female BALB/c mice were randomly divided into four groups (10 mice for each group): DREP-GP group, DREP-VP40 group, DREP-GP+DREP-VP40 group, and the negative control DREP-eGFP group. Each mouse was injected with 10 μg plasmid DNA in 100 μl PBS (10 μg DREP-GP+10 μg DREP-VP40 for DREP-GP+DREP-VP40 group) via the intramuscular route followed by immediate electroporation with ECM830 electroporation system (BTX) at the injection sites. Electroporation protocol was performed according to a previous study (Johansson et al., 2012), and consisted of 2 pulses of 1.125 V/cm for 50 ms, and 8 pulses of 275 V/cm for 10 ms. All groups were boosted with the same dose of DNA at 2 and 4 weeks. Blood samples were collected at 1 week prior to the first immunization and 2 weeks after each immunization, and serum samples were collected and stored at -80°C until further analysis.

### Determination of Anti-Ebola-Specific Antibody

The titers of IgG antibodies were measured in the serum sample from each group of mice by an enzyme-linked immunosorbent assay (ELISA). Briefly, the recombinant GP and VP40 proteins were individually coated as the antigens at a concentration of 1 μg/ml in 96-well polystyrene microtiter plates for overnight at 4°C. After blocking with 5% milk in PBS buffer, the ELISA plates were incubated with serial dilutions of serum samples at 37°C for 2 h. The titer of total IgG was detected with horseradish peroxidase (HRP)-conjugated goat against mouse IgG (1:2000 dilution, Santa Cruz Biotechnology). IgG subtype antibodies including IgG1, IgG2a, and IgG2b (1:2000 dilution, Santa Cruz Biotechnology) were further detected individually. The immune complex was developed with 3,3′,5,5′-tetramethylbenzidine (TMB) (Sigma). The reaction was stopped with 2 mol/L H_2_SO_4_, and the plates were read with the absorbance at 450 nm in a microplate reader (Bio-Rad). All samples were detected in triplicate wells.

### Cytokine Assay

The spleens from each group of mice were collected 2 weeks after the final immunization, and the splenocytes were isolated by using mouse lymphocyte separation medium (Dakewe Biotech, China). Next, 5 × 10^5^ cells were added in each well of the 96-well plates, and cultured with recombinant GP or VP40 proteins (10 μg/mL) for 72 h. Culture media were taken as the controls. The concentration of IFNγ, IL-2, IL-4, and IL-10 in the culture supernatants was measured by ELISA kits (BioLegend, United States) following the manufacturer’s procedures. All assays were performed in triplicate.

### IFN-γ ELISPOT Assay

The peptide epitopes of EBOV GP and VP40 proteins that are specific for CD8^+^ T lymphocytes in BALB/c mouse (GP-T1: LYDRLASTV; GP-T2: GPCAGDFAF; VP40-T1: YFTFDLTALK; VP40-T2: TSPEKIQAIM) were selected according to previous reports (Warfield et al., 2005; Wu et al., 2012), and synthesized (GeneScript, Nanjing, China). After the preparation of single cell suspensions from mouse splenocytes, ELISPOT assays were performed in pre-coated 96-well plates (Dakewe Biotech, China). The antibody-coated plates were blocked with complete RPMI 1640 medium for 2 h at room temperature. After blocking, 100 μl of splenocytes suspension (1 × 10^6^ cells/ml) containing different peptides (10 μg/ml) were added to each well. A positive control PMA/ionomycin (Sigma) and a media negative control were included in all assays. The plates were incubated for 24 h in a humidified incubator at 37°C, 5% CO_2_. Plates were then washed and processed according to manufacturer’s instructions, and spots were enumerated using an ImmunoSpot reader and ImmunoSpot software (Cellular Technology Ltd.). Peptide-specific CD8^+^ T-cell frequency was expressed as Spots forming cells (SFCs)/1 × 10^5^ splenocytes. Background spots (negative control wells) were subtracted from test wells. A positive response to a peptide was defined as having > 5 SFCs/1 × 10^5^ splenocytes after subtraction of the background.

### Statistical Analysis

Statistical significance between two means was assessed by using the unpaired Student’s *t*-test and a *p*-value < 0.05 was considered significant, included in the text and figures. Statistical analyses were generated using Graph Pad software.

## Results

### Expression of EBOV GP and VP40 Proteins by SFV DREP Vector

**FIGURE 1:**
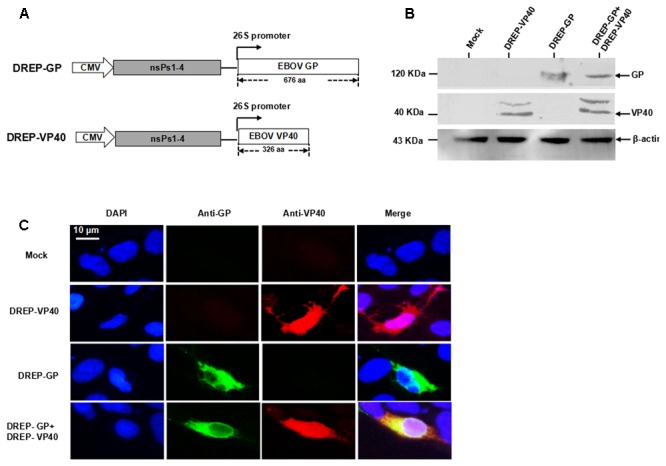
Expression of EBOV GP and VP40 proteins based on SFV DREP vectors. **(A)** Diagram of constructed recombinant DREP-GP and DREP-VP40 plasmids, nsPs represent the non-structural proteins; **(B)** Western blot; and **(C)** Indirect immunofluorescence analysis of GP and VP40 proteins in transfected 293 cells at 48 h post-transfection. β-actin was taken as the internal control in Western blot.

The genes coding the ZBOV GP and VP40 proteins were separately cloned into the downstream of the 26S subgenomic viral promoter in the DREP vector to generate the recombinant DREP-GP and DREP-VP40 vectors (**Figure 1A**). After sequence verification, 293 cells were transfected with the recombinant plasmids DREP-GP, DREP-VP40, or DREP-GP+DREP-VP40. Western blot analysis of DREP-GP or DREP-VP40 transfected cells verified the specific protein bands for GP (about 120 KDa) and VP40 (about 43 KDa) (**Figure 1B**), and their co-expression was likewise verified in 293 cells transfected with DREP-GP+DREP-VP40 (**Figure 1B**). The immunofluorescence analysis further confirmed the expression of GP and VP40 proteins in transfected 293 cells (**Figure 1C**). These results indicated that GP and VP40 could be efficiently expressed in mammalian cells using the SFV-based replicon DREP vector.

### Generation of Ebola VLPs in Cells Transfected with DREP-Based GP and VP40 Vectors

**FIGURE 2:**
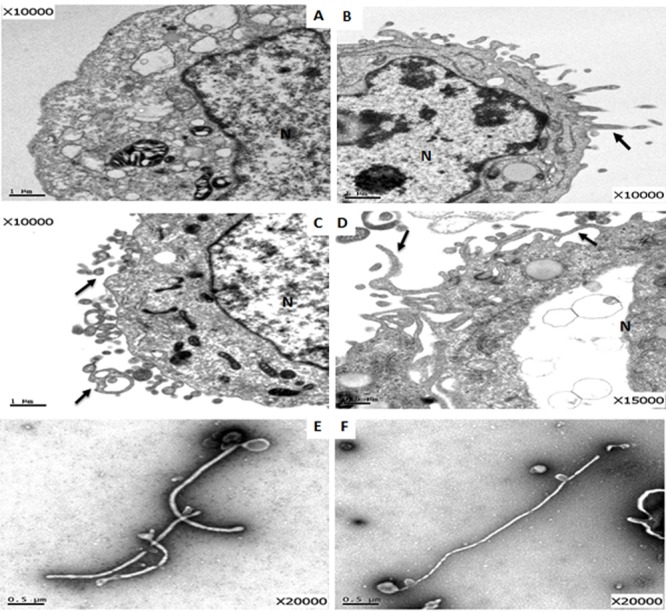
TEM analysis of the EBOV VLP assembly in DREP-based GP and VP40 transfected cells. **(A–D)** TEM analysis of ultrathin sections of cells: **(A)** 293 cells control; **(B)** 293 cells transfected with DREP-VP40 for 48 h; **(C)** 293 cells transfected with DREP-GP for 48 h; **(D)** 293 cells co-transfected with DREP-GP and DREP-VP40 for 48 h; **(E)** and **(F)** the concentrated preparations from the supernatant of cells co-transfected with DREP-GP and DREP-VP40 for 48 h. “N” represents the cell nucleus. The black arrow indicates the assembly of VLPs.

To investigate the assembly of Ebola VLPs, following transfection with DREP-based GP and VP40 plasmids for 48 h, 293 cells were fixed, sectioned, and examined under TEM. In comparison with untransfected 293 cells (**Figure 2A**), the budding of VP40-derived filamentous particles was observed in the cellular membrane of cells transfected with DREP-VP40 (**Figure 2B**). Budding of GP-derived particle structures from the cell surface was also detected in cells transfected with DREP-GP (**Figure 2C**). Additionally, TEM analysis revealed that transfection with DREP-GP+DREP-VP40 led to the assembly and budding of EBOV VLPs (**Figure 2D**). Finally, we detected the EBOV filamentous particles in the purified preparations from the supernatant of cells co-expressing GP and VP40 proteins (**Figures 2E,F**). These filamentous particles were about 80 nm in diameter and 1,500–4,000 nm in length, which were similar in size and morphology to the virus particles observed in EBOV infected cells. This finding indicated that expression of GP and VP40 using DREP vectors could generate different sizes of particle structures, including the typical filamentous structure of EBOV VLPs.

### Specific Antibody Responses Induced by Vaccination with DREP-Based GP and VP40 Vectors

**FIGURE 3:**
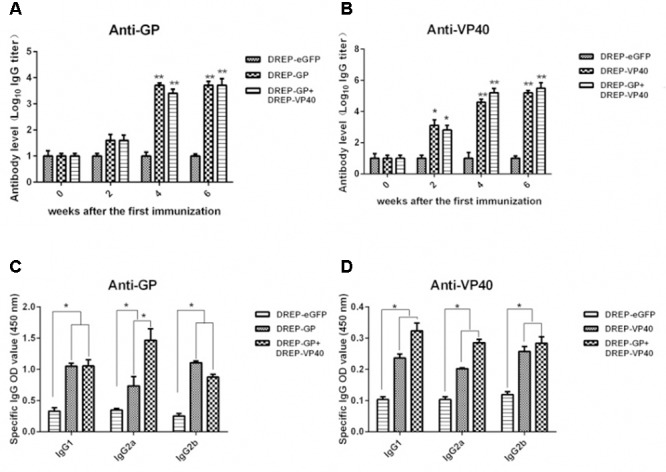
Specific antibody responses induced by immunization with DREP-based GP and VP40 vectors. **(A)** and **(B)** Determination of specific anti-GP **(A)** and anti-VP40 **(B)** antibodies in the sera of BALB/c mice collected at 0, 2, 4, and 6 weeks after the first immunization. The antibody levels were expressed as the log_10_ IgG titers. **(C)** and **(D)** IgG subtype analysis of the specific anti-GP **(C)** and anti-VP40 **(D)** antibodies derived from the sera of immunized BALB/c mice. Serum samples from mice immunized with DREP-eGFP were used as the negative control. Results were expressed as the mean of OD450 ± SEM. The results presented are representative of three independent experiments. ^∗^*p* < 0.05, ^∗∗^*p* < 0.01.

The Antibody titers were measured to test the humoral immune response induced by the DREP-based GP and VP40 vectors in BALB/c mice. After vaccination twice, the anti-GP IgG antibody titers were significantly increased in DREP-GP and DREP-GP+DREP-VP40 vaccinated mice (**Figure 3A**). Vaccination with DREP-GP+DREP-VP40, which produced Ebola VLPs containing GP and VP40 antigens *in vitro*, did not enhance the total anti-GP IgG levels compared with using DREP-GP vector alone. However, a high titer of anti-VP40 IgG antibodies was induced by vaccination with either DREP-VP40 or DREP-GP+DREP-VP40 vectors at 2 weeks after the first immunization (**Figure 3B**). Neither GP-specific nor VP40-specific antibody responses were detected in DREP-eGFP vaccinated mice. Remarkably, we detected a much higher level of IgG antibody against VP40 antigen than GP antigen in DREP-GP+DREP-VP40 vaccinated mice after the third immunization (**Figures 3A,B**).

We further analyzed the EBOV antigens–specific IgG subtypes, including IgG1, IgG2a, and IgG2b. The ELISA results showed that IgG antibodies against GP or VP40 were mainly of a mixed IgG subtype in BALB/c mice vaccinated with DREP-based GP and VP40 vectors including DREP-GP, DREP-VP40, and DREP-GP+DREP-VP40 (**Figures 3C,D**). Interestingly, we found that DREP-GP+DREP-VP40 induced a higher titer of anti-GP IgG2a subtype antibodies than the DREP-GP alone (**Figure 3C**). However, there was no significant difference in the induction of anti-VP40 antibody subtypes between the DREP-GP+DREP-VP40 and DREP-VP40 vectors. The above results suggested that DREP-based GP and VP40 vectors elicited GP and VP40-specific antibodies in mice, and that co-immunization of DREP-GP+DREP-VP40, which generated EBOV VLPs *in vitro*, significantly enhanced the level of IgG2a antibody to the GP antigen.

### Cytokine Production by Vaccination with DREP-Based GP and VP40 Vectors

**FIGURE 4:**
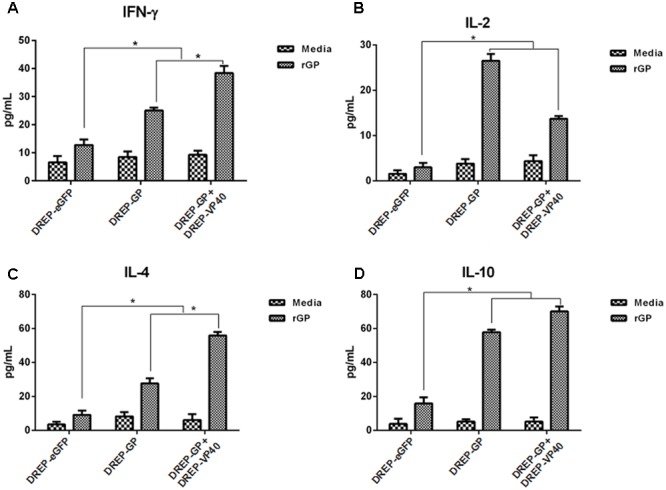
ELISA analysis of cytokines secreted by splenocytes from immunized mice upon stimulation with EBOV recombinant GP proteins (rGP). **(A–D)** Determination of the cytokines IFN-γ **(A)**, IL-2 **(B)**, IL-4 **(C)**, and IL-10 **(D)** production in the supernatant of splenocytes by ELISA 2 weeks after the final immunization. The results for three independent experiments are shown as the mean ± SD. ^∗^*p* < 0.05.

**FIGURE 5:**
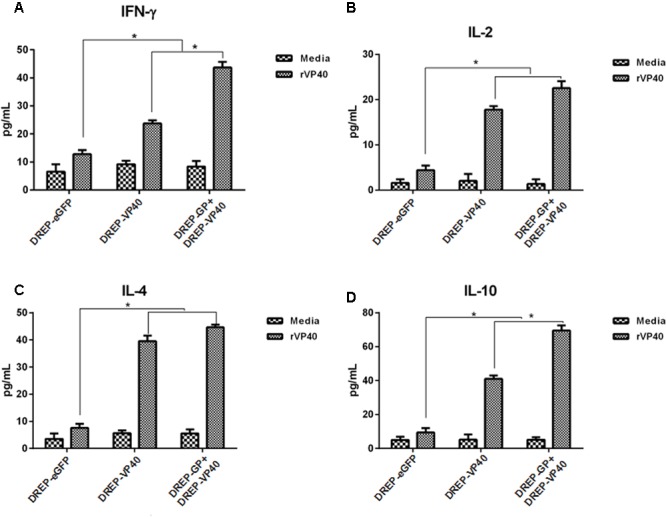
ELISA analysis of cytokines secreted by splenocytes from immunized mice upon stimulation with EBOV recombinant VP40 proteins (rVP40). **(A–D)** Determination of the cytokines IFN-γ **(A)**, IL-2 **(B)**, IL-4 **(C)**, and IL-10 **(D)** production in the supernatant of splenocytes by ELISA 2 weeks after the final immunization. The results for three independent experiments are shown as the mean ± SD. ^∗^*p* < 0.05.

To investigate the cytokines characteristic of T-cell responses following DREP-based GP and VP40 vaccination, the splenocytes were stimulated *in vitro* with recombinant GP (rGP) or VP40 (rVP40) proteins for 3 days, using culture media alone as the negative control. The supernatants from the cultured splenocytes were detected for IFN-γ, IL-2, IL-4, and IL-10. As shown in **Figure 4**, splenocytes from both DREP-GP and DREP-GP+DREP-VP40 immunized mice secreted higher levels of IFN-γ, IL-2, IL-4, and IL-10, relative to DREP-eGFP control, upon stimulation with rGP (*p* < 0.05). Furthermore, immunization with DREP-GP+DREP-VP40 significantly elicited the secretion of IFN-γ and IL-4 over DREP-GP alone (*p* < 0.05). Similarly, when stimulating with rVP40, an increased secretion of IFN-γ, IL-2, IL-4, and IL-10 cytokines was observed in the splenocytes derived from both DREP-VP40 and DREP-GP+DREP-VP40 immunized mice (**Figure 5**). Finally, DREP-GP+DREP-VP40 vectors enhanced the secretion of IFN-γ and IL-10 compared with DREP-VP40 alone (*p* < 0.05). The results suggested that DREP-based GP and VP40 vectors induced a mixed T-cellular immune response, including both Th1 and Th2 cytokines. More importantly, co-immunization with DREP-based GP and VP40 vectors elicited higher levels of IFN-γ production than vaccination with DREP-GP or DREP-VP40 alone.

### Generation of Ebola-Specific CD8^+^ T Lymphocyte Responses

**FIGURE 6:**
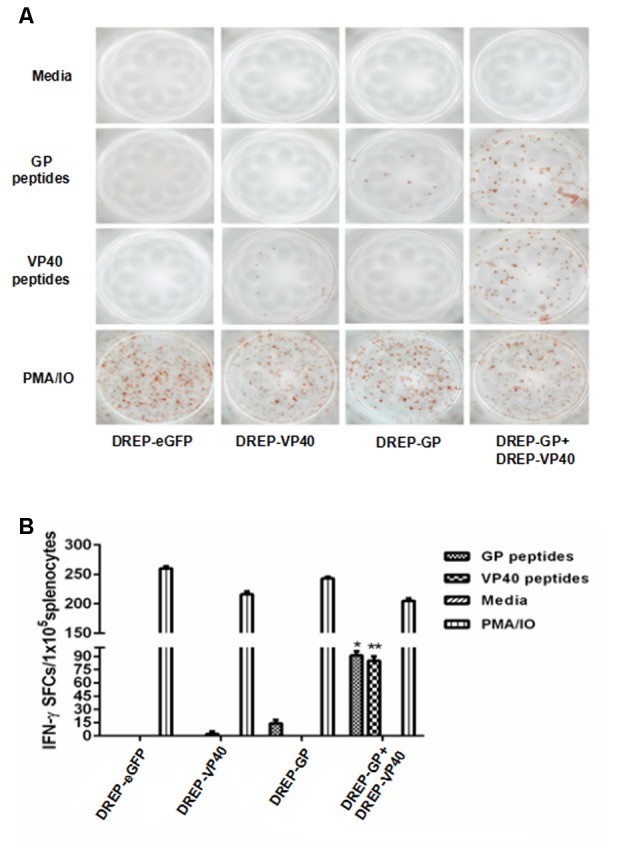
ELISPOT analysis of IFN-γ secreting CD8^+^ T cells from immunized mice upon stimulation with GP- and VP40-specific epitopes. Splenocytes were isolated from plasmid-immunized mice and were stimulated *in vitro* with GP- or VP40-specific epitopes. A positive control (PMA/IO) and a negative control (Media) were included. IFN-γ-specific spot forming was observed **(A)**, and the numbers of IFN-γ SFCs/1 × 10^5^ splenocytes were counted **(B)**. The results for three independent experiments are shown as the mean ± SD. ^∗^*p* < 0.05; ^∗∗^*p* < 0.01.

The IFN-γ ELISPOT assay was performed to examine the Ebola antigen-specific CD8^+^ T lymphocyte responses. Splenocytes were collected and stimulated with GP-derived or VP40-derived CD8^+^ T-cell-specific peptides for IFNγ production. The splenocytes stimulated with PMA/Ionomycin or culture media were used as the positive control, or the negative control, respectively. As shown in **Figure 6A**, there were an average of 14 GP-specific and 6 VP40-specific IFNγ secreting CD8^+^ T cells (per 1 × 10^5^ splenocytes), which were derived from DREP-GP and DREP-VP40 immunized mice, correspondingly. Notably, we detected an average of 91 GP-specific and 85 VP40-specific IFNγ secreting CD8^+^ T cells (per 1 × 10^5^ splenocytes) in DREP-GP+DREP-VP40 immunized mice (**Figures 6A,B**). Neither GP- nor VP40-specific IFNγ secreting CD8^+^ T-cell responses were generated in the DREP-eGFP control group. These results demonstrated that co-vaccination with DREP-based GP and VP40 antigens elicited EBOV-specific CD8^+^ T lymphocyte responses, which were superior to vaccination with DREP-GP or DREP-VP40 alone.

## Discussion

In this study, we employed an Alphaviruses SFV replicon-based DNA vector DREP to express EBOV GP and VP40 antigens. Recently, several Alphavirus replicon vectors such as SFV and Venezeulan equine encephalitis virus (VEEV) have been extensively evaluated as new kinds of vaccine vectors in pre-clinical and clinical immunogenicity studies (Knudsen et al., 2012; Wecker et al., 2012; Bates et al., 2016). For example, DNA-launched replicons (DREP) from SFV that express the HIV T-cell immunogen have resulted in a strong induction of CD8^+^ T-cell responses in mice, which requires only nanogram quantities of the replicon DNA (Knudsen et al., 2012). Furthermore, Alphavirus vectors provide the ability for the replicating RNA to stimulate a strong type I interferon (IFN) response and to initiate apoptosis in the transfected target cell (Tonkin et al., 2010; Knudsen et al., 2012). More importantly, the Alphavirus replicon-encoding DNA vaccines can break immunological tolerance by activating innate antiviral pathways, which may provide a strategy for overcoming the relatively weak immunogenicity of conventional DNA plasmids, while avoiding the side effects of highly immunogenic viruses or strong adjuvants (Leitner et al., 2003).

The expression of GP was confirmed in 293 cells transfected with DREP-GP and DREP-GP+DREP-VP40. EBOV GP is a type-1 transmembrane protein that is cleaved into N-terminal GP1 subunit and C-terminal GP2 in the Golgi complex, and presented on the virion envelope as a homotrimeric spike (Mohan et al., 2015). There is also secretory GP (sGP) produced by RNA editing that forms a sGP-GP2 complex and confers infectivity (Iwasa et al., 2011). Cells expressing GP produce different sizes of particle-like structures budding from the plasma membrane (Noda et al., 2002). In our study, the similar particle-like virosomes with different shapes and diameters were also detected in the 293 cells transfected with DREP-GP. Furthermore, the assembly and budding of filamentous particles was observed in the 293 cells transfected with DREP-VP40. EBOV matrix protein (VP40) regulates viral budding and structure as well as virus stability (Bornholdt et al., 2013). The sole expression of VP40 is sufficient to generate filamentous EBOV VLPs, but the VLP production is poor (Johnson et al., 2006). However, co-expression of VP40 and other viral proteins, especially GP, NP, and/or VP24, can enhance the production and release of VLPs by approximately 40-fold (Licata et al., 2004). We found that the filamentous EBOV particles were efficiently released from the plasma membrane after co-transfection of DREP-GP and DREP-VP40. Moreover, the EBOV filamentous VLPs were observed in the supernatant of cells expressing GP and VP40 together rather than alone, suggesting that co-expression of GP and VP40 is likely to be essential for the production of Ebola VLPs.

Our results showed that immunization with DREP-GP- and DREP-GP+DREP-VP40-based vectors could induce similar levels of anti-GP-specific IgG antibodies in mice. More specifically, when compared with DREP-GP alone, DREP-GP+DREP-VP40 immunization enhanced the titer of anti-GP IgG2a subtype antibody, which has proven to be critical in protecting mice from EBOV challenge (Wilson et al., 2000). The enhanced titer may be due to the co-expression of VP40 antigens because the enhanced levels of IgG2a antibody are induced by VLPs containing both GP and VP40 compared with the GP vesicle preparation in an insect cell-derived system (Ye et al., 2006). Immunization with DREP-GP+DREP-VP40, however, induced similar titers and subtypes of anti-VP40 antibodies relative to DREP-VP40 immunization alone. Although the role of anti-VP40 antibodies in protection remains unknown, EBOV-infected individuals mount a strong antibody response to VP40, suggesting that humoral responses directed against several key epitopes of VP40 may contribute to protecting humans against EBOV infection (Becquart et al., 2014).

The cellular immune response is associated with the production of several cytokines including IFN-γ and interleukins. Our results demonstrated that vaccination of DREP-GP+DREP-VP40 induced higher levels of IFN-γ and interleukin (IL)-4 as well as similar levels of IL-10 compared with vaccination of DREP-GP. Moreover, compared with vaccination of DREP-VP40, vaccination of DREP-GP+DREP-VP40 induced higher levels of IFN-γ and IL-10 as well as similar levels of IL-4. These results indicated that DREP-GP+DREP-VP40 induced a mixed Th1/Th2 cellular immune response. The importance of Th1 and Th2 responses in mediating protection against lethal EBOV infection has been identified in several reports (Olinger et al., 2005; Warfield et al., 2005; Geisbert et al., 2008). For example, the EBOV VLPs vaccination does not provide any protection for βδ TCR-deficient or CD8^+^ T-cell-deficient mice from EBOV challenge (Olinger et al., 2005; Warfield et al., 2005). Additionally, the VSV-based GP vaccinated animals with the lowest CD4^+^ T-cell counts succumb to lethal ZEBOV challenge (Geisbert et al., 2008). Interestingly, we also observed a different IL-2 response after vaccination of DREP-GP or DREP-VP40 alone compared with co-vaccination of DREP-GP and VP40, which may be worth further investigation, given that IL-2 is important for the immune cell proliferation.

In this study, immunization with DREP-GP+DREP-VP40 could enhance the generation of IFN-γ secreting CD8^+^ T cells compared to immunization with DREP-GP alone. This result may be explained by the ability of GP antigens to efficiently induce DC maturation and generation of IFN-γ^+^ T cells with the co-expression of VP40 compared with GP alone (Warfield et al., 2003; Ye et al., 2006). We also found that DREP-GP+DREP-VP40-immunized mice produced more IFN-γ secreting T cells upon stimulation with the VP40 protein or epitopes than DREP-VP40 immunization alone, and showed no significant difference in IL-2 and IL-4 secretion. This finding suggests that the VP40 elicits T-cell responses in addition to humoral immunity, but inclusion of the GP protein may be required to induce sufficient protective responses (Lehrer et al., 2017). A future protection experiment would be essential to evaluate the efficiency of DREP-based EBOV-vector vaccine against EBOV infection.

## Conclusion

We have successfully generated EBOV GP and VP40 antigens based on SFV DREP vectors. The high titers of specific IgG antibodies accompanied with a mixed Th1/Th2 cellular immune response were detected in the mice vaccinated with DREP-based GP and VP40 vectors. Our study provides an alternative vaccine strategy to produce EBOV antigens for Ebola disease prevention.

## Author Contributions

WL and SL conceived and designed the study. SR, QW, LC, and CX performed the experiments. WL, SR, QW, and XY analyzed the data and drafted the manuscript. FT and JL instructed the procedures and revised the manuscript critically. All authors read and approved the final manuscript.

## Conflict of Interest Statement

The authors declare that the research was conducted in the absence of any commercial or financial relationships that could be construed as a potential conflict of interest.
